# Editorial: CNS autoimmune disorders and COVID-19

**DOI:** 10.3389/fneur.2023.1183998

**Published:** 2023-04-04

**Authors:** Omid Mirmosayyeb, Shervin Badihian, Vahid Shaygannejad, Hans-Peter Hartung

**Affiliations:** ^1^Department of Neurology, Jacobs Comprehensive MS Treatment and Research Center, Jacobs School of Medicine and Biomedical Sciences, University at Buffalo, State University of New York, Buffalo, NY, United States; ^2^Neurological Institute, Cleveland Clinic, Cleveland, OH, United States; ^3^Department of Neurology, School of Medicine, Isfahan University of Medical Sciences, Isfahan, Iran; ^4^Department of Neurology, Medical Faculty, Heinrich Heine University Düsseldorf, Düsseldorf, Germany; ^5^Brain and Mind Centre, University of Sydney, Sydney, NSW, Australia; ^6^Department of Neurology, Medical University of Vienna, Vienna, Austria

**Keywords:** coronavirus disease, COVID-19, CNS - central nervous system, CNS autoimmune disorders, severe acute respiratory syndrome coronavirus 2 (SARS-CoV-2)

Acute autoimmune disorders involving the central nervous system (CNS) are a group of diseases that occur when the immune system attacks and damages brain and spinal cord cells and tissues. neuromyelitis optica spectrum disorder (NMOSD), myelin oligodendrocyte glycoprotein antibody disease (MOGAD) and multiple sclerosis (MS) are some examples of CNS autoimmune disorders (1). COVID-19 is a highly contagious respiratory disease caused by the severe acute respiratory syndrome coronavirus 2 (SARS-CoV-2) and belongs to the Coronaviridae family.

Since late 2019, COVID-19 has been spreading globally and has affected all people around the world (2). COVID-19 may increase the risk of developing neurological symptoms, such as headaches, confusion, ageusia, and anosmia (3), as well as some neurological disorders, like encephalopathy, stroke, seizures, hypoxic/ischemic brain injury, and a number of CNS autoimmune diseases (4). On the other hand, COVID-19 infection can deteriorate the pre-existing neurological diseases in affected individuals (5, 6).

With respect to CNS autoimmune disorders, several reports have been published on individuals developing different forms of autoimmune encephalitis following COVID-19 infection (7). These include patients with anti-*N*-Methyl-D-Aspartate Receptor encephalitis, anti-Myelin oligodendrocyte glycoprotein (MOG) antibody encephalitis, acute disseminated encephalomyelitis (ADEM), as well as other variants of autoimmune encephalitis (7, 8). Moreover, COVID-19 has been shown to cause demyelinating diseases of CNS in a number of reports (8).

Additionally, acute inflammatory demyelinating polyneuropathy (AIDP) is one of the commonly reported autoimmune diseases after COVID-19 infection (9). Guillain-Barré syndrome (GBS) is probably the most frequent subtype of AIDP reported among these patients and presents with muscle weakness, paralysis, and impairments in coordination and balance which could have devastating outcomes if not treated urgently (8). Other forms of polyneuropathy have also been reported among affected individuals (8).

Lastly, COVID-19 has been shown to exacerbate preexisting neurological conditions (5, 6). The immune response to the virus may further worsen the symptoms of conditions such as multiple sclerosis, Alzheimer's disease, Parkinson's disease, epilepsy, and stroke probably in the setting of increased inflammation (6, 7). Specifically, with regards to multiple sclerosis, this is explained by alterations in the T cell counts during the disease course as well as fluctuations in body temperature that can worsen some neurological symptoms in these patients (6).

Another relevant area that has been investigated is the susceptibility of individuals suffering from neurological diseases or taking immunosuppressive/immunomodulatory medications to COVID-19 and their disease outcome. Patients taking anti-CD20 medications, which deplete B-cells, may be more prone to contract COVID-19 although it may not necessarily increase rates of hospitalization (9).

COVID-19 can affect the central nervous system through a variety of routes (10). In some individuals, SARS-CoV-2 may trigger an overactive immune response of Th1, Th2, NK cell, DC, and elevated levels of pro-inflammatory cytokines of IL-1β, IL-2, IL-6, IL-8, IL-12, IL-17 that results in the development of autoantibodies against the CNS. These autoantibodies can attack the protective myelin coating of the nerves, resulting in inflammation and damage. In addition, the olfactory bulb is another route that SARS-CoV-2 can pass, cross the blood-brain barrier (BBB), and infect the CNS (10). Even though there is increasing evidence linking COVID-19 and the autoimmune diseases of the CNS, more research is required to better understand the mechanisms that underlay this relationship and to determine whether or not COVID-19 contributes to the development of autoimmune diseases of the CNS.

The development of vaccines against COVID-19 has been the most important tool to fight the COVID-19 pandemic and decrease the disease severity, hospitalization rates, and mortality (11). However, there are some reports on neurological complications of these vaccines (12). The majority of these complications are mild and transient, such as headaches, while a small number of people may develop more serious side effects (13). These include cerebral sinus venous thrombosis, Bell's palsy, transverse myelitis, and GBS (14). Of note, the evidence on these complications comes mostly from case reports which do not provide strong evidence regarding this association (14).

In this issue, eleven interesting studies have presented that focus on CNS autoimmune diseases and COVID-19 infection, as outlined below.

Lotan et al. investigated the risk of CNS demyelinating diseases following COVID-19 infection through a systematic review. They showed that the risk of developing these diseases or experiencing relapses in the setting of COVID-19 infection remains relatively low with a favorable outcome. Elizalde-Díaz et al. further explained the relationship between inflammatory and humoral immune markers activated through COVID-19 infection and their effect on neural cells and subsequent neurological complications seen among some patients.

Czarnowska et al. reported on the safety of the COVID-19 vaccine among patients with MS on disease-modifying therapies and found overall favorable outcomes with low risk. On the other hand, three case studies reported on autoimmune complications of COVID-19 vaccines, including a case of multiple autoimmune syndromes in Poli et al. study, a case of immune thrombotic thrombocytopenia with cerebral venous thrombosis and hemorrhage in Chen et al. study, a case of acute disseminated encephalomyelitis by Bastide et al., and a patient with NMO reported by Ghelmez et al.. Moreover, Rinaldi et al. reported six patients with CNS inflammatory demyelinating events (two acute transverse myelitis, three multiple sclerosis, and one NMOSD) following COVID-19 infection.

The humoral response to COVID-19 vaccines and immunogenicity among patients with pre-existing CNS autoimmune disorders was assessed by three studies. Dominelli et al. showed that disease-modifying therapies, specifically depleting/sequestering-out treatments, lower the humoral response to COVID-19 vaccines, while cellular responses are still achieved. Similarly, van Dam et al. looked at the humoral response to the vaccine among patients with MS who had contracted COVID-19 before, and found increased humoral responses in patients without anti-CD20 therapies, but decreased responses among those treated with ocrelizumab. Lastly, Sedaghat et al. studied a group of patients with multiple sclerosis who had remained seronegative following two doses of inactivated COVID-19 vaccines and suggested adenoviral vector or mRNA-based vaccines may be a better choice as the third dose in these cases.

In conclusion, the current evidence demonstrates how the COVID-19 pandemic has led to the development of CNS autoimmune diseases such as MS, NMOSD, autoimmune encephalitis, and AIDP. These complications, however, remain relatively infrequent despite the large number of people affected by COVID-19. On the other hand, patients with pre-existing neurological disorders are affected, both with deterioration/relapse of their symptoms and with the increased risk of developing a more severe infection in the setting of immunosuppressive/immunomodulatory therapies. Of note, the current evidence on this topic is still limited and warrants further studies on larger populations with prospective designs. Lastly, the COVID-19 vaccine has shown to be safe and very effective in decreasing disease contraction, severity, and mortality, although it rarely can lead to CNS autoimmune disorders. The vaccination strategies among patients on disease-modifying therapies is another challenging topic that requires further investigation.

## Author contributions

All authors listed have made a substantial, direct, and intellectual contribution to the work and approved it for publication.
